# Spatial Video Health Risk Mapping in Informal Settlements: Correcting GPS Error

**DOI:** 10.3390/ijerph16010033

**Published:** 2018-12-24

**Authors:** Andrew Curtis, Sandra Bempah, Jayakrishnan Ajayakumar, Dania Mofleh, Lorriane Odhiambo

**Affiliations:** 1 GIS Health & Hazards Lab, Kent State University, Kent, OH 44242, USA; sbempah@kent.edu (S.B.); jajayaku@kent.edu (J.A.); 2 College of Public Health, Kent State University, Kent, OH 44242, USA; dmofleh@kent.edu (D.M.); lodhiamb@kent.edu (L.O.)

**Keywords:** GIS, GPS, spatial video, geonarratives, slum, informal settlement, WASH

## Abstract

Informal settlements pose a continuing health concern. While spatial methodologies have proven to be valuable tools to support health interventions, several factors limit their widespread use in these challenging environments. One such technology, spatial video, has been used for fine-scale contextualized mapping. In this paper, we address one of the limitations of the technique: the global positioning system (GPS) coordinate error. More specifically, we show how spatial video coordinate streams can be corrected and synced back to the original video to facilitate risk mapping. Past spatial video collections for the Mathare informal settlement of Kenya are used as an illustration as these data had been previously discarded because of excessive GPS error. This paper will describe the bespoke software that makes these corrections possible, and then will go on to investigate patterns in the coordinate error.

## 1. Introduction

Informal settlements (IS) pose a considerable global challenge as they are the sites of multiple health, environmental, and social problems. At the same time, they are constantly changing and are data poor. To counter this spatial data deficiency, public health or epidemiological initiatives often rely on global positioning system (GPS) support. One example of a geospatial technology that has been used in challenging environments is spatial video (SV). SV combines imagery with locational information and can be used as a digitizing source for both cross-sectional and longitudinal research [1,2,3]. One problem influencing SV use is the GPS error caused by the typical type of IS built environment. These errors are especially problematic as SV requires spatial precision when mapping the video. If the coordinate connected to a video frame is wrong, so too will be the final digitized map.

This paper will show how problematic GPS coordinate paths can be corrected and then synced to the original video. This is not an improvement of the actual GPS signal, or even an automated search for error, but rather an SV-specific post-processing procedure. To illustrate this, we will use SV data collected over a three-year period for the Mathare IS in Kenya. 

### 1.1. Mapping Challenging Environments

IS mapping occurs for a variety of reasons including establishing legitimacy [4], identifying tenancy, and guiding official renewal or improvement efforts [5,6]. Spatial support has also been used in health or epidemiological investigations [7] and the associated identification of environmental risk layers, for example, the impact of flooding during the rainy season. Data sources and techniques range from community involvement and participatory mapping [8] to remotely sensed data (satellite and aerial imagery, even from unmanned aerial vehicles) [9,10], citizen sensor/volunteered geographic information (VGI) [8], and census collections. Each mapping approach has both benefits and limitations [11]. For example, a census can provide valuable household insights, but the logistics required typically make this cross sectional, which is not ideal in such a changing landscape. While satellite and especially high-resolution aerial photography is more easily acquired, processed, and used to dynamically update an IS, the closely packed roof lattice makes individual home and on-the-ground features difficult to extract [4]. VGI offers both community empowerment and the chance to update features [12], but problems include oversight, quality control, and, in the case of the Kibera IS in Nairobi, Kenya, even competing platforms [13]. 

Mapping IS environments is critical for many reasons, for example addressing the area’s high disease burden [9,14]. Intervention teams require guidance as how best to target limited resources as these landscapes are far from homogenous with risks varying by season [15], time of day [16,17], and by micro-environments. For example, access to clean water, the availability of adequate sanitation, and appropriate personal hygiene (water and sanitation for health or WASH) [18,19,20] are constant challenges in an IS. WASH is also spatial, with the proximity to drainage or toilets and the intersection with elevation (acquired from DEM data) being useful to predict where disease is most likely to occur [7]. SV provides one solution for mapping IS environments [1,21,22] as the output allows the user to know exactly where each frame was captured. In this way, features such as drainage channels, latrines, standing water, mud, trash, animals, and human activity can be mapped, all of which can have implications in terms of local disease risk [23]. SV can also provide detail on the spaces between buildings, along the ground where children play, and the quality of the environment around each water point or toilet. A spatial video geonarrative (SVG), which adds an interview recorded at the same time as the SV, can further contextualize these spatial layers [24]. SVG can explain not only where health risks occur but also why they exist and persist. For example, if safety concerns affect utilization of WASH locations then SVG can help explain the location and usage of routes/water points/toilets and identify which are problematic. However, one challenge has doggedly hindered handheld SV collected in IS environments. These environments cause considerable GPS error and signal dropouts. Sometimes simplistic manipulations are applied such as substituting a straight-line (Euclidean) distance between features, which, if scaled accordingly, has been shown to be appropriate in more homogenous settings [25]. However, SV cannot use such a simple solution because the method relies on the linking of an image with an actual coordinate.

Research in the developed world has considered similar GPS problems, for example, in association with accelerometers [26]. Issues such as the subject being stationary or moving or what happens when a building is entered [27] can all lead to incomplete or unreliable coordinate streams. Solutions have included either discarding the data or imputing between two reliable coordinates [28]. Other studies have focused more on the GPS unit’s performance and how to systematically identify and correct for signal error [29], such as through calculating a moving centroid [30]. While these GPS limitations are well described, IS environments pose additional challenges, including satellite availability, intervening obstacles (especially the nature of the built environment), and the quality of the receiver. IS settlements such as Mathare often comprise of narrow corridors with overhanging roofs made of metal that can cause signal reflection [31]. Compounding this is the danger in such environments; therefore, the researcher tends to use a smaller, less powerful GPS unit that is sometimes hidden by the researcher when people are near. The result is often a spaghetti of coordinate paths that vary wildly from known routes when mapped.

Solutions beyond simple imputation have included recreating activity paths using supporting data, such as photographs, video, or recall [31]. For SV use, this is not an option as the GPS signal needs to be corrected and linked back to the original video stream. If this can be achieved, then tasks such as identifying the same toilet or buildings and environment around it are made possible. 

In this paper, we return to archived SV collected in the Mathare IS of Nairobi and show how bespoke software can correct and even create coordinate paths that can be linked back to the original video. We describe the process of how this correction occurs before using the newly created SV to explore the location and extent of the original GPS error.

### 1.2. Mathare IS, Kenya

The Mathare IS is located within Nairobi County, Kenya, and along with the Kibera IS, present two of the oldest and most health-challenged settlements in Kenya. Limited access to safe water, unsanitary practices including open defecation and trash dumping, high human density often in close proximity to disease-causing environmental factors (such as standing water), and generally unsafe living conditions all occur in the IS [32]. In Mathare, enteric diseases, pneumonia, diabetes, HIV, acute respiratory infections, bronchitis, and the recent emergence of malaria occur frequently, disproportionately impacting women and children [14,33]. Several studies have collected valuable social (and to some degree spatial) data relevant to WASH in Mathare, such as water points, latrines, drainage channels, and dumpsites [14]. In the Map Kibera project, which also includes Mathare, young people mapped drainage channels using GPS devices supported with satellite imagery and on-the-ground photos. These data have also been enriched with additional surveys or interviews, while geographic information system (GIS) buffering has been used to estimate the number of households using each toilet or the number of people per tap [14]. 

Mapping water points, water fetching routes, human activity and domiciles, localized water flow, and other micro-environmental risks could all be vital in preventing disease in Mathare [7,34]. Attribute detail connected to these features could include water point availability [9], water quality, and even an index of safety, especially for women [35,36]. Added into this could be the proximity of homes to open drainage [25,37,38] and where water (and then mud) settles [31,39,40,41], and also the location of toilets and their type, whether they are clean, where they drain, and whether they are emptied or prone to overflow during rains. While Mathare has been the focus of considerable health research [14,32,42], and though it has been mapped in a variety of different ways [15], this type of complex spatial and temporal mapping has largely been absent. Conceptually, with frequent SV collection, we should be able to improve these maps dynamically with updatable localized risks.

## 2. Materials and Methods

Spatial video transects were collected by a local researcher for the Mathare slum between 2013 and 2015 using a hand-carried Contour + 2 camera. This is a simple to use and robust video camera designed for extreme sports use. The camera contains an internal GPS receiver, which means locational information is attached to each second of video. The initial goal of data collection was to map fine-scale health-related environmental risks across different time periods. All SVs were either uploaded through the cloud or delivered by carrier to the GIS Health and Hazard Lab at Kent State University. An initial assessment of these videos found that the quality of the GPS paths varied dramatically due to the physical conditions within Mathare. As a result, it was not possible to use these as a digitizing resource and these data were discarded for approximately two years. With advances in spatially supported programming tools, these SVs were again reassessed in 2017 for the purposes of mapping. 

The GPS was extracted from each usable video, meaning there was useful content lasting at least 30 seconds. These GPS coordinates were input into the bespoke software that allows for the creation of a new (corrected) path, which could be synced back to the original video. This new SV could then be used as a source to digitize risks in the same software using a Google Maps interface. The GPS correction software, which was built on freely available software services including Google Maps API, YouTube API, and the JavaScript framework Data-Driven Documents (D3), uses a linear spatial interpolation algorithm to create the new GPS path. To add imagery to help the correction, the YouTube Iframe API was used to embed videos to webpages with JavaScript being used to control the YouTube player in the webpage. 

**Figure 1:**
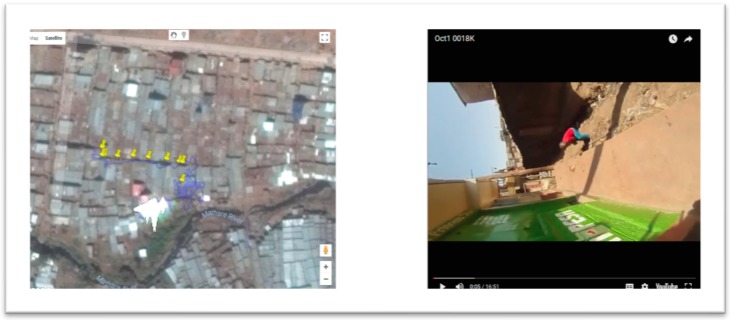
The software interface of the coordinate correction software; the left side shows the original global positioning system (GPS) path with yellow pins being used to create the new path. The image on the right is the spatial video (SV), and as can be seen, the image is sometimes challenging to work with.

Figure 1 displays the interface of the coordinate correction software video player; the left-side window maps the GPS path that corresponds with the video playing in the right-side window. To correct the GPS path, the output coordinate file was used as a reference because in most instances the original SV still contained accurate sections that could guide the correction process. A consideration in designing the GPS correction and risk mapping software was to make it as ubiquitous as possible, both in terms of user ease and data transfer. To this end, all SVs are uploaded to YouTube (using a non-public URL) as this reduces file sizes allowing for easier data transfer between collaborators.

As has been previously mentioned, the issue of GPS coordinate dropouts is well known. However, Contour Storyteller software (which is the supporting software of the SV camera) masks these omissions by imputing between the coordinates on either end of this dropout. The resulting path in Contour Storyteller does not distinguish between actual coordinates and the imputed path. Our new SV player accounts for these errors by color coding actual GPS points and filling between the coordinate gaps with an interpolated line.

The user views the video and matches it with the extracted GPS path. When this GPS path wanders from where it should go (ascertained by comparing the video and overhead imagery), the user then manually corrects the coordinates. This is done by using easily discernible landmarks such as notable buildings, empty spaces, water bodies, vegetation, roads (and turns), bridges, and rivers/drainage canals. Pin markers are manually dropped onto the map when these features are seen on the YouTube video, with the software adding the associated media time stamp from the video as an attribute to the pin. This same approach can be used to backtrack from a GPS coordinate to “fill in” sections of the path where there had been no coordinates. For example, in Figure 1, the GPS path starts approximately 5 minutes after the beginning of the video. The software is used to create coordinates for the beginning five minutes by reversing the video and again using landmarks to guide pin placement. In this example, these features include the alley where the walk started, as well as open drains close to a bridge, vegetation especially single bushes, and proximity and orientation to some of the few high-rise buildings around Mathare. 

More specifically, each pin (called a track point) is encoded with both a location attribute and an associated time stamp from the video; location <x,y> at video time t seconds will have the information as a triplet of the form <x,y,t>. A linear interpolation algorithm is used to generate spatiotemporal triplets between two track points that are separated in space and time. For two spatiotemporal triplets of the form <x_1_,y_1_,t_1_> and <x_n_,y_n_,t_n_>, the linear interpolation algorithm generates the series <x_2_,y_2_,t_2_>, <x_3_,y_3_,t_3_>, …, <x_n−2_,y_n−2_,t_n−2_>, <x_n−1_,y_n−1_,t_n−1_>. Therefore, if we have two track points with a data tuple of the form <x_1_,y_1_,t_1_> and <x_k_,y_k_,t_20_> and there is a temporal difference of 20 seconds, the algorithm calculates the slope for the line between <x_1_,y_1_> and <x_k_,y_k_> and uses this information to generate 20 points between <x_1_,y_1_> and <x_k_,y_k_>. The assumption of slope being constant works for straight segments but tends to be erroneous when there are sharp turns. To counter this, or on generally non-regular paths, the user provides additional reference points as needed.

**Figure 2:**
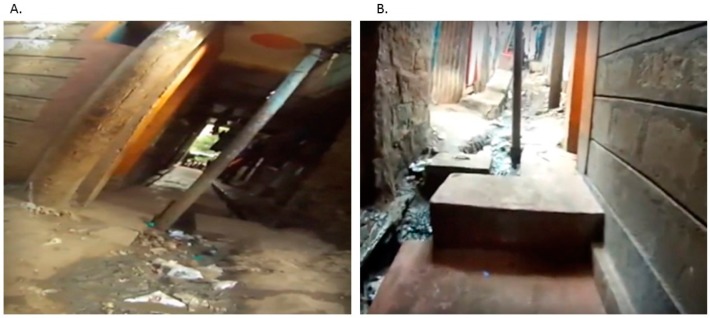
The point of intersection between two paths. (**A**) is from a video taken on 19 March 2014 and (**B**) on 8 October 2015. (**A**) was taken from the opposite direction with respect to (**B**). Features used to validate both path’s correct coordinate location include the orange painted wall, the pole, the striped pattern on the wall, and the drain.

After GPS correction, the new path is downloaded as a GPX GPS file. The video can then be synced back to the GPS as a JSON file, in effect creating a new SV where the video progresses simultaneously with a cursor on the map. This JSON file has a small data footprint and can easily be emailed between collaborators. Validation of the new data can occur by matching newly digitized locations to secondary data sources, such as independently corrected GPS locations. Alternatively, this can be achieved where two different SV paths intersect at the same location, such as when routes are collected at different times (Figure 2). 

## 3. Results

**Figure 3:**
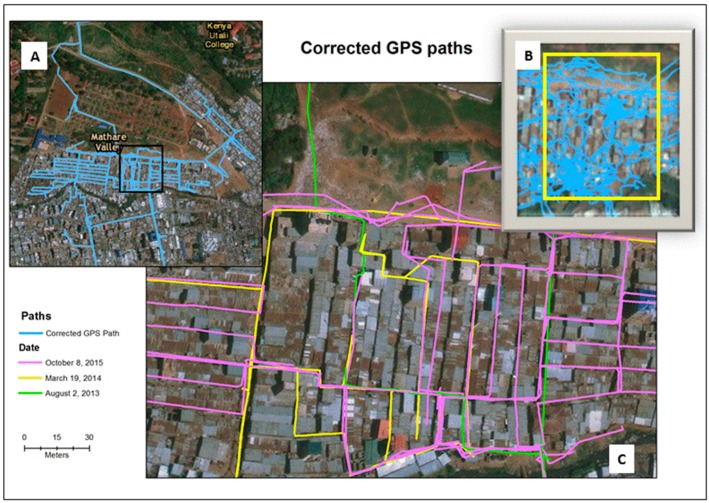
A map showing all corrected GPS paths for Mathare (**A**), with corrected paths from three time periods (**C**) that coincide with the box in (**A**). (**B**) shows the uncorrected GPS tracks for this same area.

During the period August 2013 to October 2015, 26 handheld SVs were collected in the Mathare informal settlement of Kenya. All of these SV coordinate paths were improved using the GPS correction software. Figure 3 displays the corrected GPS paths for the entire study neighborhood (A), and a detail of three time periods (C) that coincides with the box on the inset map. The uncorrected paths (B) can also be compared with the same area. The SV within the residential area of Mathare was more easily corrected than the open areas where there were fewer visual cues. This graphic also shows various path intersections, which can be used for environmental change comparisons and GPS path validations.

**Figure 4:**
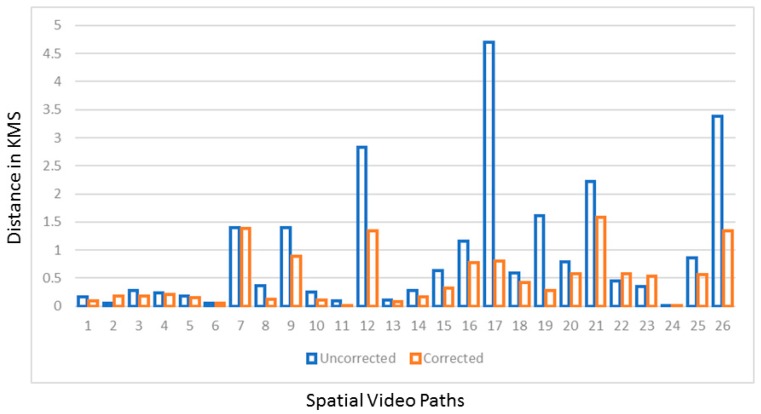
The variations in lengths of paths before and after correction. In most instances the original GPS path is shorter (in four cases less than half the length). This variation is mostly due to the signal bouncing between surfaces leading to a distance overestimation. The few instances where the corrected path is longer (22 and 23) are because the software allowed for the re-creation of the GPS path where the original signal was lost.

In previous research, GPS paths in an IS have been used to gain insights into different activities, such as water fetching. However, the physical distance as calculated by just using uncorrected GPS paths could lead to both over- (because the signal bounces so much) and underestimation (due to missing sections of coordinates). To determine the degree of this error, we calculated the differences in distance between each of the corrected and uncorrected paths. The amount of overestimation is considerable, with 85% of the corrected paths being less than 1 km long as compared to 69% of the uncorrected paths. Figure 4 shows this graphically for each path.

**Figure 5:**
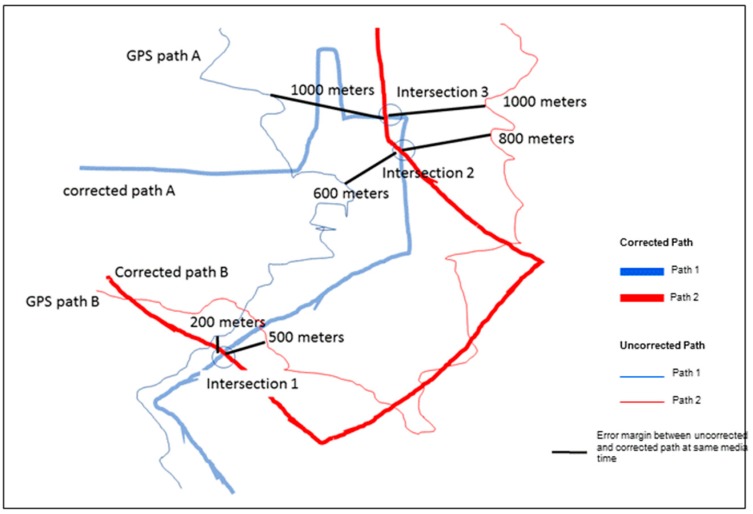
A conceptualization of the error between corrected and uncorrected coordinates for two different SV paths.

An alternative approach to visualize this error is to map the differences between the corrected and uncorrected paths at every coordinate. As the SV progresses, each frame has a corresponding coordinate and, therefore, we can measure the distance between the frame coordinate of the uncorrected and corrected paths. Figure 5 illustrates this conceptually showing two different time periods and for two corrected and uncorrected paths. Three intersection points were used to display the amount of error involved. For example, at intersection 1, there was a difference of 200 meters between the corrected and uncorrected paths of route A and 500 meters for route B.

**Figure 6:**
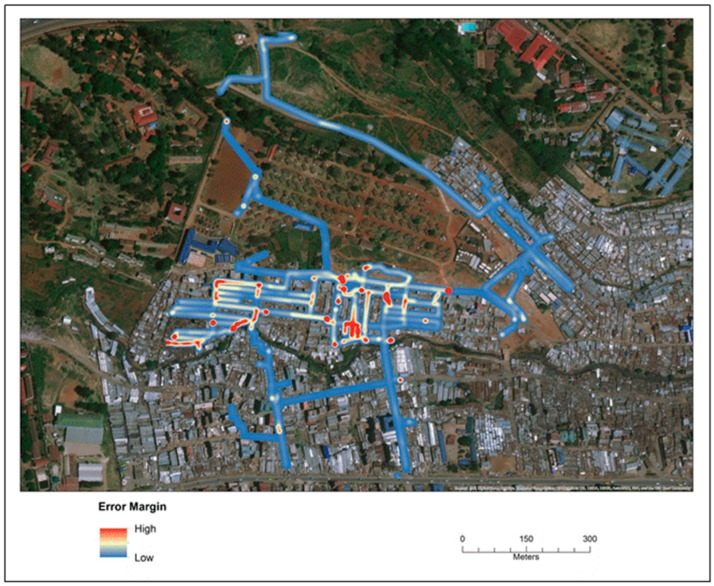
A hot spot map showing intensities of error, meaning the distance between the actual and corrected location for all coordinates along each SV path.

The error for each path was then visualized using a kernel density estimation (KDE), which is a common technique applied in epidemiological studies to show variations in spatial intensity. In this case the error for every corrected coordinate on all the SV routes was combined and used as input into the KDE. Figure 6 displays where the error was consistently higher across all SVG routes in Mathare for all time periods. 

**Figure 7:**
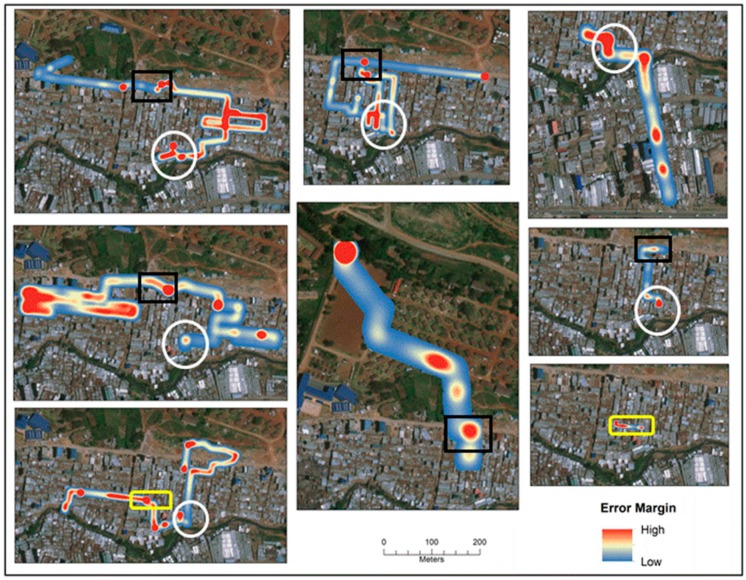
Visualized error between actual and corrected coordinate paths for eight SV routes. The three shapes show the same location on the different maps.

To further consider the geographic pattern of this error, Figure 7 displays eight different routes so that the same sections can be compared.

**Figure 8:**
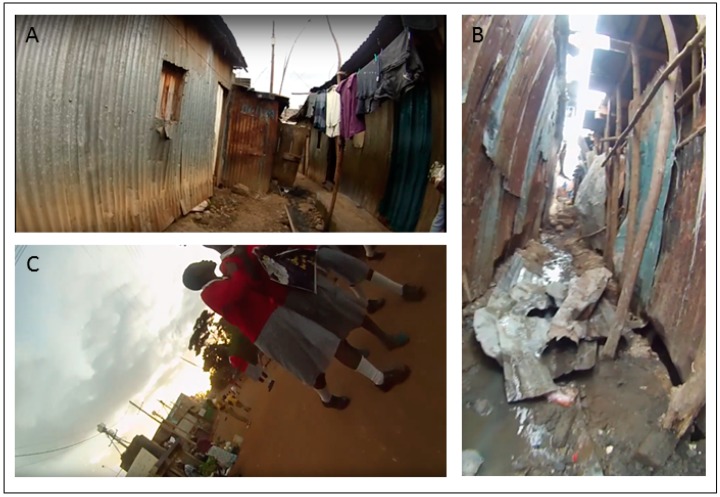
Example images from the three areas of comparison mapped in Figure 7 (white circle = (**A**), yellow rectangle = (**B**), black square = (**C**)).

Figure 8 shows example images taken from the SV for the areas mapped in Figure 7. The area around A is comprised of tight alleyways but with some open space. This area is closer to the Mathare River. Area B is more densely packed in terms of metallic buildings latticed with tight alleys. Area C is a dusty road with open space around it. It is heavily used by motorists and pedestrians with considerable roadside vendor activity. Both Areas A and B show stability in the mapped error. This was somewhat expected given the nature of the landscape with tight corridors and closely packed structures made of highly reflective material which can affect the GPS signal. Fewer such physical impediments appear within C, though this can also contain the most human activity of the three example locations. This means that the camera was concealed more frequently for security reasons, which led to problems with the GPS signal. This last point may also help explain why there is variable error for the same section over different time periods.

## 4. Discussion

IS neighborhoods continue to pose one of the greatest global health challenges. Yet, an important public health intervention tool having updateable, fine-scale mapping remains largely elusive in these environments. As health resources tend to be limited, having this type of spatial support is essential. It is vital to know where health risks are greatest, how these risks change over time, and where patients reside and how best to reach them. Spatial video and geonarrative collection provide an exciting opportunity to remedy this. The low cost and relative ease of use of these techniques also makes longitudinal collection possible, which is vital for capturing these ever-changing environments. An achievable goal using these technologies would be a comprehensive spatial characterization of health risks; for example, which water points are safest at any time period. Risks could also be identified around the home, along known pathways, and in the activity/play spaces of children. In other words, we can map a complex environment, dynamic in nature, contextualized with insights that not only explain why risks occur but also how local response strategies have developed. In this way, newly settled families could, for example, be warned about and then told how to deal with externalities, such as flooding and the human waste it brings into the home.

This type of near real time mobile geospatial support is not wishful thinking. There are already localized mapping initiatives used by residents and activist groups wanting to improve the quality of existence in Kenyan IS. As SV cameras become even smaller and more powerful—the new generation of police body-worn cameras being a good example—this technique could become a standard part of a researcher or health official’s toolkit. A further benefit would be to bring local community involvement into the process through data collection and knowledge creation. In so doing, existing online maps showing key features could be continuously updated with more (contextualized) detail [8].

As one example, the previously described Map Kibera crowdsourcing group have also produced an online map of Mathare. While the online Mathare map covers a much larger area than that described in this paper, there is still an overlapping section containing eight toilets and six water points. SV paths used in this paper intersected with six of the toilets, though only four were visible on the SV (one for two different time periods). All six of the water points fell on SV paths with four being visible in the video. Of these, one water point was visible for three different time periods and another for two. It is not hard to imagine how SV updates could regularly update this map. They could even provide additional detail through images, mapping of the surrounding environment, and narratives of local concerns. By adding in biological testing, such as water point quality, a dynamic web map could be created that would inform both residents and professionals about their best water choices in near real time. This type of quality control map would be essential for the wellbeing of residents, especially during disease outbreaks. 

However, an impediment to such SV use in IS environments has traditionally been the poor quality of the GPS coordinates. While traditional GPS use can potentially be improved by using aerial photography to drop a point at the correct location, SV use requires a more complicated correction procedure that links the new coordinate stream back to the original imagery. In this paper we have shown how correction is possible, meaning that past and future SV imagery can now be used to map out health risks and document fine-scale change. In addition, the same software allows for basic mapping, meaning that the combination of GPS correction, risk mapping, and online availability of the videos make this a ubiquitous IS resource.

The paper has also shown geographic patterns in the original coordinate error. While these are not surprising (tight corridors with multiple metallic surfaces will cause GPS error), they do reveal two considerations that are important for future work. First, even with more powerful GPS units, these errors, though reduced in intensity, are still likely to occur. Secondly, it should be possible to develop automation procedures to correct GPS coordinate paths. By knowing the coordinates of key features (ascertained from previous SV paths), time differences that span error hotspots could be used to impute the correct SV coordinate path. Currently, this assumes the SV path goes in the same direction and at the same speed but as more pathway overlays are collected, so more reference points are added and direction becomes unimportant. Eventually, a grid of correctly referenced features and the paths connecting them would only require the SV to be snapped into place with far less manual involvement. While this next step is being worked on, the correction approach described in this paper means that IS initiatives should already consider using SV for localized mapping and health promotion. 

### Limitations

There are several limitations to the implementation of the coordinate correction software. Firstly, the video needs to be uploaded to YouTube, which can be problematic if internet speeds are slow. The authors are currently working with NSF funding to develop new spatial video compression techniques that will improve this problem. Once uploaded, however, Internet speed is less important, with the biggest issue then being able to view high definition YouTube videos. Secondly, the accuracy measure of the newly corrected coordinate path is dependent on three things: the effort of the user, the quality of the overhead imagery, and the type of environment. If the user is dedicated, has access to high-resolution overhead imagery (which can be used (in combination with the video) to identify where the path goes), and the landscape is relatively heterogeneous, then there is no reason why the path cannot be accurately recreated. Thirdly, while this software is free and available from the authors on request, it is not currently available for download on a public website with technical support. Until such a time, the authors welcome any request for access to the software or code.

Issues regarding inter-rater reliability, the effort required to turn SV into usable maps, and the ethics and privacy issues regarding spatial video use have been covered in several prior publications [23,43,44].

## 5. Conclusions

In this paper, we have shown that new spatial software can correct or even create GPS paths that can be synced back to the original video. This allows for existing SV collections to be repurposed, and even ordinary video spatialized through the geocoding of key features on the video. As a result, SV can now be used to update existing maps, create better fine-scale contextualized geographic data for research and health intervention, and generally make available the types of health risk mapping that have not been commonly available in these types of environments.
